# Significance of mesothelin and CA125 expression in endometrial carcinoma: a retrospective analysis

**DOI:** 10.1186/s13000-021-01093-4

**Published:** 2021-04-08

**Authors:** Soichiro Kakimoto, Morikazu Miyamoto, Takahiro Einama, Yasuhiro Takihata, Hiroko Matsuura, Hideki Iwahashi, Hiroki Ishibashi, Takahiro Sakamoto, Taira Hada, Jin Suminokura, Tsubasa Ito, Rie Suzuki, Ayako Suzuki, Masashi Takano

**Affiliations:** 1 Department of Obstetrics and Gynecology, National Defense Medical College Hospital, Tokorozawa, Saitama 359-8513 Japan; 2 Department of Surgery, National Defense Medical College Hospital, Tokorozawa, Saitama 359-8513 Japan; 3 Department of Host Defense and Biochemical Research, Juntendo University Graduate School of Medicine, Tokyo, 113-8431 Japan

**Keywords:** Endometrial carcinoma, Mesothelin, CA125, Prognosis, Co-expression, Gynecologic carcinoma

## Abstract

**Background:**

This study aimed to investigate the association between clinicopathologic factors, mesothelin, and cancer antigen (CA) 125 in endometrial carcinoma.

**Methods:**

Between 1989 and 2017, patients with endometrial carcinoma who underwent total hysterectomy and bilateral salpingo-oophorectomy at our hospital were identified. The association between either or both immunochemical expression of mesothelin and CA125 and clinicopathological features were retrospectively examined.

**Results:**

Among 485 patients, 171 were positive for mesothelin, 368 were positive for CA125, and 167 were positive for mesothelin and CA125. The expression of mesothelin and CA125 was positively correlated (*p* < 0.01). More patients with mesothelin expression showed myometrial invasion of more than 50% (*p* = 0.028) and positive lymphovascular invasion (*p* = 0.027). Similarly, more patients with co-expression of mesothelin and CA125 had myometrial invasion of more than 50% (*p* = 0.016) and positive lymphovascular invasion (*p* = 0.02). Patients with mesothelin expression and co-expression of mesothelin and CA125 demonstrated worse progression-free survival (PFS) and overall survival (OS). In the multivariate analysis, mesothelin expression and co-expression were poor prognostic factors for PFS (mesothelin expression: hazard ratio [HR] = 2.14, *p* < 0.01; co-expression: HR = 2.19, *p* < 0.01) and OS (mesothelin expression: HR = 2.18, *p* < 0.01; co-expression: HR = 2.22, *p* < 0.01).

**Conclusions:**

Mesothelin expression and co-expression might be associated with tumor aggressiveness and poor prognosis in patients with endometrial carcinoma. Persons with mesothelin-expressing endometrial cancers present a particularly high medical unmet need.

## Background

The incidence of endometrial carcinoma (EC) has gradually increased, and ECs is one of the major types of gynecologic carcinomas [1]. The standard treatments for EC are surgery and adjuvant therapy including chemotherapy and radiotherapy according to the recurrent risk factor [2]. Due to the advances in treatment, the prognosis of these patients has dramatically improved, but those with advanced disease stage of aggressive histological subtypes such as serous carcinoma showed worse prognosis [3].

Mesothelin, a 40-kDa protein, is normally expressed in mesothelial cells of the pleural cavity, peritoneal cavity, and peritoneum [4, 5]. Furthermore, mesothelin has a strong affinity to cancer antigen 125 (CA125) with N-linked glycans [6]. Several studies reported that co-expression of mesothelin and CA125 correlated with aggressive features of tumors and poor prognosis of several carcinomas such as ovarian carcinomas [7–16]. However, only a few studies have investigated the association between mesothelin and CA125 expression in ECs [17–20]. Therefore, we considered the need to study the relationship between these expressions and clinicopathological features.

Herein, this study aimed to evaluate the correlation between the clinicopathological factors and either the expression of mesothelin or CA125 or co-expression of these biomarkers in endometrial carcinoma.

## Methods

### Patients

Patients with endometrial carcinoma who underwent total hysterectomy and bilateral salpingo-oophorectomy in our hospital between 1989 and 2017 were identified. All patients were re-evaluated pathologically according to the 2020 World Health Organization criteria [21]. Those with grades 1, 2, and 3 endometrioid carcinoma, serous carcinoma, clear cell carcinoma, carcinosarcoma, and mixed carcinoma were included in this study. Patients with other histological types, complicated with other carcinomas, and lack of either clinical information or surgical specimens were excluded. All patients qualified in the period were included. The clinicopathological factors were obtained from the medical records.

### Immunohistochemical analysis

Four-hundred and eighty-five formalin-fixed paraffin-embedded tissues were used for tissue microarray (TMA). All slides were stained immunohistochemically as previously reported [20]; anti-mesothelin (clone 5B2 diluted 1:50 Leica: NCL-L-MESO) and anti-CA125 (clone M11 diluted 1:50 DAKO:M3520) antibodies were used under the same conditions.

### Immunohistochemical evaluation

**Fig. 1:**
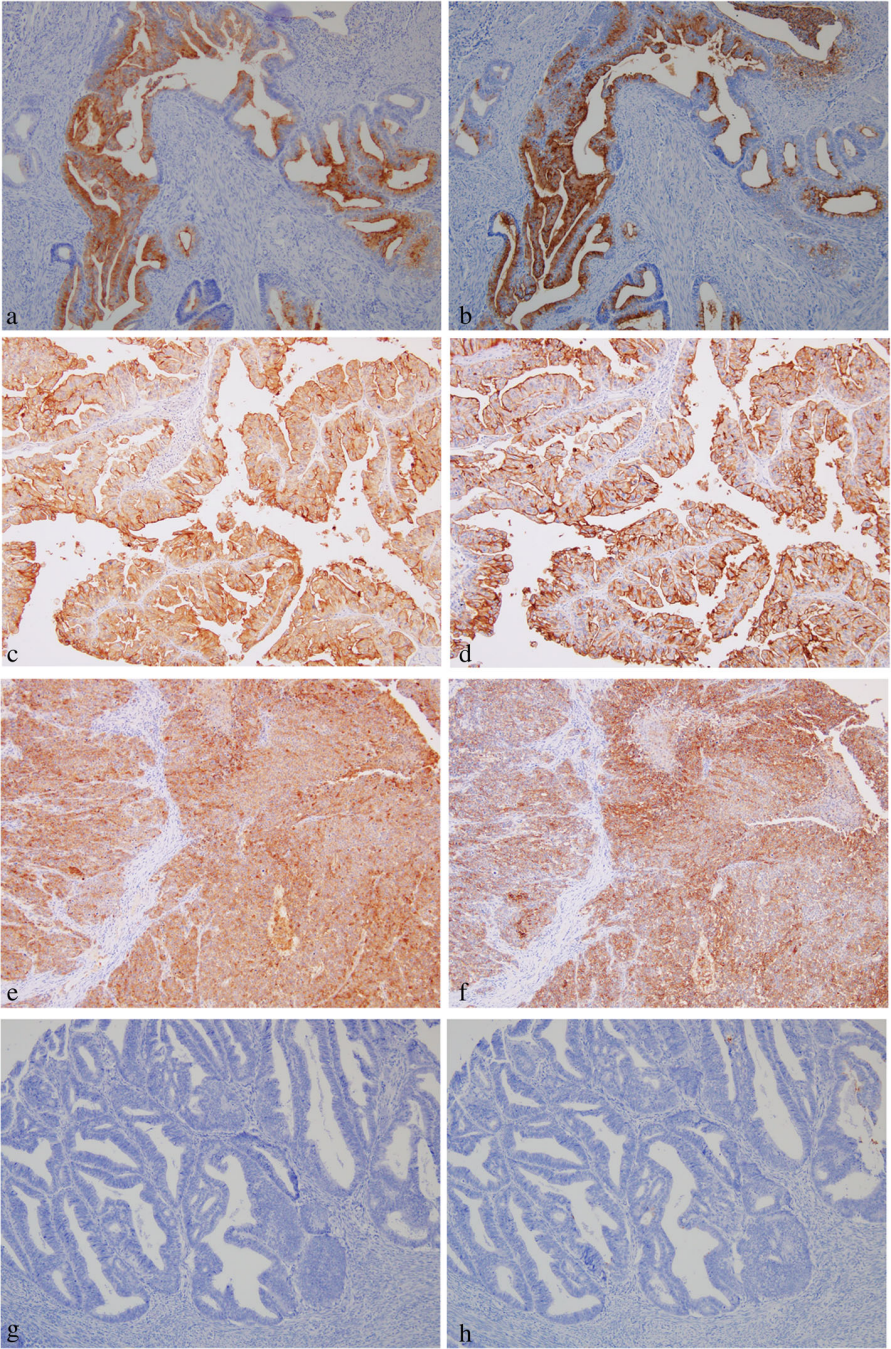
The expression variation of mesothelin and cancer antigen (CA) 125. **a** Mesothelin-positive cells in grade 1 endometrioid carcinoma. The percentage of mesothelin-positive cells is ≥50% (tumor proportion score: 3+), and the entire circumference of the cancer cell membrane is stained (intensity score: 2+). **b** CA125-positive cells of grade1, endometrioid carcinoma. The percentage of CA125-positive cells is ≥50% (proportion score: 3+), and the cytoplasm of cancer cell is highly stained (intensity score: 2+). **c** Mesothelin-positive cells in grade 2 endometrioid carcinoma. The percentage of mesothelin-positive cells is ≥50% (tumor proportion score: 3+), and the entire circumference of the cancer cell membrane is stained (intensity score: 2+). **d** CA125-positive cells of grade2, endometrioid carcinoma. The percentage of CA125-positive cells is ≥50% (proportion score: 3+), and the cytoplasm of cancer cell is highly stained (intensity score: 2+). **e** Mesothelin-positive cells in grade 3 endometrioid carcinoma. The percentage of mesothelin-positive cells is ≥50% (tumor proportion score: 3+), and the entire circumference of the cancer cell membrane is stained (intensity score: 2+). **f** CA125-positive cells of grade3, endometrioid carcinoma. The percentage of CA125-positive cells is ≥50% (proportion score: 3+), and the cytoplasm of cancer cell is highly stained (intensity score: 2+). **g** Mesothelin-negative cells in grade 1 endometrioid carcinoma. No staining is noted in the cancer cell’s membrane. **h** CA125-negative cells in grade 1 endometrioid carcinoma. No staining is noted in the cancer cell’s membrane. Magnification for all slides: × 100. *HE, hematoxylin-eosin

The evaluation methods used were those described in prior literature and our previous studies [10, 20]. The expression of mesothelin and CA125 was determined by evaluating the tumor proportion score and staining intensity score. The tumor proportion score, defined as the percentage of mesothelin- or CA125-positive cells in carcinoma tissues, was as follows:0, no tumor cells stained in the entire carcinoma tissue; 1+, ≥1 to < 10% of cells stained in the entire carcinoma tissue; 2+, ≥10 to < 50% of cells stained in the entire carcinoma tissue; and 3+, ≥50% of cells stained in the entire carcinoma tissue. The staining intensity score was defined as follows: 0, no tumor cells stained in the entire carcinoma tissue; 1+, incomplete membrane staining and/or faint or barely perceptible cytoplasmic staining detected; and 2+, entire circumference of the cell membrane stained and/or moderate to strong cytoplasmic staining. A tumor proportion score of 3+ and/or a staining intensity score of 2+ was considered positive for mesothelin (Fig. 1a) or CA125 expression (Fig. 1b). On the contrary, other cases were considered negative for mesothelin (Fig. 1c) or CA125 expression (Fig. 1d). Additionally, presence of both mesothelin-positive and CA125 cells was considered as co-expression. Two observers evaluated immunoreactivity without prior information of clinical data. In the interpretation of immunohistochemistry, any discrepancies between 2 observers were resolved through discussion over a multiviewer microscope.

### Statistical analysis

Histological subtypes were classified into four types: grade 1, endometrioid carcinoma; grade 2, endometrioid carcinoma; grade 3, endometrioid carcinoma, and other carcinomas including serous carcinoma, clear cell carcinoma, carcinosarcoma, and mixed carcinoma. The χ^2^ test and Fisher’s exact test were used to confirm the correlation between the clinicopathological data and mesothelin and CA125 expression. Progression-free survival (PFS) and overall survival (OS) curves were drawn using the Kaplan-Meier method. The differences between the survival curves were analyzed using the Cox proportional hazard test. In the statistical analysis, all *p*-values of < 0.05 were considered significant. The software JMP® 14.0 (SAS Institution Inc., Cary, NC, USA) was used to perform all statistical analyses.

## Results

The details of the tumor proportion and intensity scores are shown in Table 1. Two-hundred and sixteen patients (45%) showed absence of mesothelin expression. A total of 68 (14%) patients had a tumor proportion score of 3+, while 166 had a staining intensity score of 2+ on mesothelin staining Among the 485 patients, 171 (35%) were positive for mesothelin expression. Similarly, 56 patients (11%) showed absence of CA125 expression. Two hundred seventy (56%) patients had a tumor proportion score of 3+, while 364 (75%) had a staining intensity score of 2+ on CA125 staining. Moreover, 368 (75%) patients showed were positive for CA 125 expression, while 167 (34%) demonstrated co-expression of mesothelin and CA 125. The relationship between the histological subtypes and expression is shown in Table 2. Only a few patients had grade 1 endometrioid carcinoma and more had other types of carcinomas in the groups with positive mesothelin expression than in those with negative mesothelin expression (*p* = 0.027). More patients had grade 1 endometrioid carcinoma in groups with positive CA125 expression than in those with negative CA125 expression (*p* < 0.01). In the groups with positive co-expression, only a fewer patients had grade 1 endometrioid carcinoma and more had other types of carcinomas than in those with negative co-expression (*p* = 0.048). Among 171 patients with positive mesothelin expression, 168 (98%) showed co-expression of mesothelin and CA125 (*p* < 0.01).

**Table 1:** Immunohistochemical analysis of mesothelin and CA125 expression in endometrial carcinoma

	Scores of different proportions of mesothelin-positive cells								Scores of different proportions of CA125-positive cells							
	0		1+		2+		3+		0		1+		2+		3+	
Staining intensity score																
0	216	(45%)	0	(0%)	0	(0%)	0	(0%)	56	(11%)	0	(0%)	0	(0%)	0	(0%)
1+	0	(0%)	58	(12%)	40	(8%)	5	(1%)	0	(0%)	33	(7%)	28	(6%)	4	(1%)
2+	0	(0%)	34	(7%)	69	(14%)	63	(13%)	0	(0%)	22	(4%)	76	(16%)	266	(55%)

*CA125* cancer antigen 125

**Table 2:** Association between histological subtypes of endometrial carcinoma and either/or co-expression of mesothelin and CA125

Histological type	Mesothelin expression					CA125 expression					Co-expression of mesothelin and CA125				
	Positive		Negative		*p*-value	Positive		Negative		*p*-value	Positive		Negative		*p*-value
	(*n* = 171)		(*n* = 314)			(*n* = 368)		(*n* = 117)			(*n* = 167)		(*n* = 318)		
Grade 1 endometrioid carcinoma	65	(38%)	155	(49%)	0.027	182	(49%)	38	(32%)	< 0.01	64	(38%)	156	(49%)	0.048
Grade 2 endometrioid carcinoma	45	(26%)	63	(20%)		80	(22%)	28	(24%)		43	(26%)	65	(21%)	
Grade 3 endometrioid carcinoma	20	(12%)	45	(15%)		37	(10%)	28	(24%)		20	(12%)	45	(14%)	
Other carcinomas	41	(24%)	51	(16%)		69	(19%)	23	(20%)		40	(24%)	52	(16%)	

Other carcinomas, carcinomas, including serous carcinoma, clear cell carcinoma, carcinosarcoma, and mixed carcinoma

**Fig. 2:**
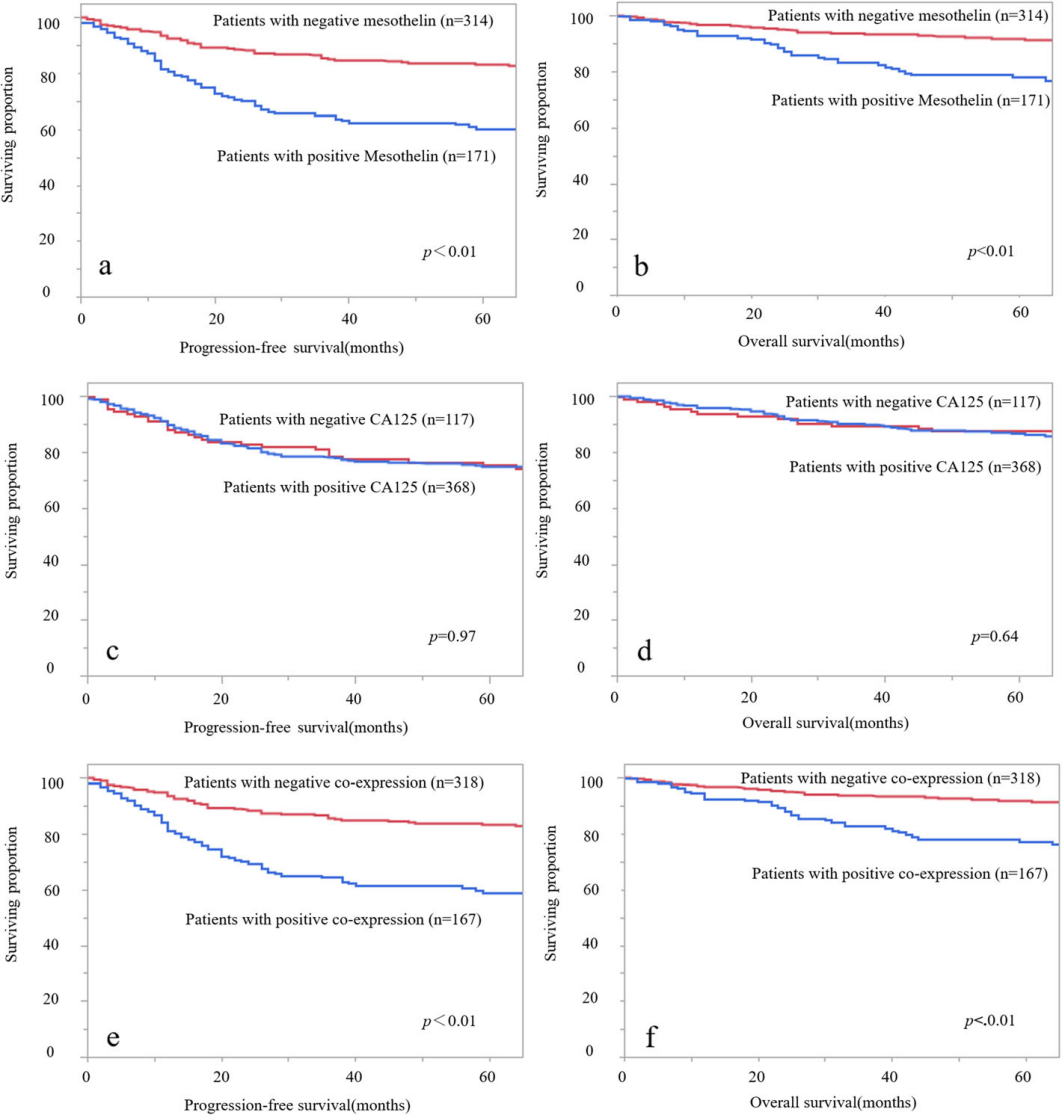
Progression-free survival (PFS) and overall survival (OS) of patients according to their differences in expression of mesothelin and CA125, and in the co-expression of both mesothelin and CA125. PFS (**a**) and OS (**b**) according to mesothelin expression, PFS (**c**) and OS (**d**) according to CA125 expression, and PFS (**e**) and OS (**f**) co-expression of both were shown

First, we investigated the association between mesothelin expression and clinical parameters. More patients in groups with positive mesothelin expression had myometrial invasion of more than 50% (*p* = 0.028) and positive lymphovascular invasion (*p* = 0.027) than those with negative mesothelin expression (Table 3). No significant correlations were observed with the other factors. Patients with positive mesothelin expression showed worse PFS and OS than those with negative mesothelin expression (Fig. 2a: PFS, *p* < 0.01; Fig. 2b: OS, *p* < 0.01). Multivariate analysis showed that positive mesothelin expression was a worse prognostic factor for PFS (Table 4: hazard ratio [HR] = 2.14, 95% confidence interval [CI] = 1.45–3.16, *p* < 0.01) and OS (Table 5; HR = 2.18, 95% CI = 1.31–3.71, *p* < 0.01).

**Table 3:** Association between pathological features and either/or co-expression of mesothelin and CA125

Parameter	Mesothelin Expression					CA125 Expression					Co-Expression of mesothelin and CA125				
	Positive		Negative		*p*-value	Positive		Negative		*p*-value	Positive		Negative		*p*-value
	(*n* = 171)		(*n* = 314)			(*n* = 368)		(*n* = 117)			(*n* = 167)		(*n* = 318)		
Age															
≥ 60	99	(58%)	153	(49%)	0.053	188	(51%)	64	(55%)	0.52	97	(58%)	155	(49%)	0.055
< 60	72	(42%)	161	(51%)		180	(49%)	53	(45%)		70	(42%)	163	(51%)	
FIGO stage															
I and II	124	(73%)	242	(77%)	0.27	280	(76%)	86	(74%)	0.62	120	(72%)	246	(77%)	0.18
III and IV	47	(27%)	72	(23%)		88	(24%)	31	(26%)		47	(28%)	72	(23%)	
Depth of myometrial invasion															
≥ 1/2	71	(42%)	99	(32%)	0.028	128	(35%)	42	(36%)	0.82	71	(42%)	99	(31%)	0.016
< 1/2	100	(58%)	215	(68%)		240	(65%)	75	(64%)		96	(58%)	219	(69%)	
Lymphovascular invasion															
Yes	85	(50%)	123	(39%)	0.027	156	(42%)	52	(44%)	0.74	84	(50%)	124	(39%)	0.02
No	86	(50%)	191	(61%)		212	(58%)	65	(56%)		83	(50%)	194	(61%)	
Cervical invasion															
Yes	27	(16%)	66	(21%)	0.18	71	(19%)	22	(19%)	1.0	27	(16%)	66	(21%)	0.27
No	144	(84%)	248	(79%)		297	(81%)	95	(81%)		140	(84%)	252	(79%)	
Ovarian metastasis															
Yes	14	(8%)	16	(5%)	0.23	24	(7%)	6	(5%)	0.66	14	(8%)	16	(5%)	0.16
No	157	(92%)	298	(95%)		344	(93%)	111	(95%)		153	(92%)	302	(95%)	
Lymph node metastasis															
Yes	27	(16%)	38	(12%)	0.35	50	(14%)	15	(13%)	0.93	27	(16%)	38	(12%)	0.30
No	111	(65%)	223	(71%)		254	(69%)	80	(68%)		108	(65%)	226	(71%)	
Unevaluated	33	(19%)	53	(17%)		64	(17%)	22	(19%)		32	(19%)	54	(17%)	
Distant metastasis															
Yes	17	(10%)	22	(7%)	0.29	27	(7%)	12	(10%)	0.33	17	(10%)	22	(7%)	0.22
No	154	(90%)	292	(93%)		341	(93%)	105	(90%)		150	(90%)	296	(93%)	
Peritoneal cytology															
Yes	37	(22%)	52	(17%)	0.17	71	(19%)	18	(15%)	0.41	37	(22%)	52	(17%)	0.17
No	134	(78%)	262	(83%)		297	(81%)	99	(85%)		130	(78%)	266	(83%)	
Adjuvant therapy															
Yes	99	(58%)	170	(54%)	0.44	201	(55%)	68	(58%)	0.52	98	(59%)	171	(54%)	0.33
No	72	(42%)	144	(46%)		167	(45%)	49	(42%)		69	(41%)	147	(46%)	

*CA125* cancer antigen 125, *FIGO* International Federation of Obstetrics and Gynecology

**Table 4:** Univariate analyses and multivariate analyses for progression-free survival in patients with endometrial carcinoma about clinicopathological features and mesothelin, CA125 expressions

Variables		Univariate analysis			Multivariate analysis for mesothelin expression			Multivariate analysis for co-expression of mesothelin and CA125		
		HR	(95% CI)	*p*-value	HR	(95% CI)	*p*-value	HR	(95% CI)	*p*-value
Age	≥ 60 vs. < 60	2.18	(1.50–3.23)	< 0.01	1.79	(1.19–2.72)	< 0.01	1.79	(1.19–2.73)	< 0.01
Histological type	Grade 2 endometrioid carcinoma vs. Grade 1 endometrioid carcinoma	3.37	(1.95–5.96)	< 0.01	2.01	(1.12–3.67)	0.019	2.02	(1.12–3.69)	0.018
	Grade 3 endometrioid carcinoma vs. Grade 1 endometrioid carcinoma	5.17	(2.88–9.36)	< 0.01	2.83	(1.49–5.42)	< 0.01	2.84	(1.50–5.45)	< 0.01
	Other carcinomas vs. Grade 1 endometrioid carcinoma	8.92	(4.41–12.6)	< 0.01	2.48	(1.34–4.70)	< 0.01	2.49	(1.35–4.72)	< 0.01
FIGO stage	III. IV vs. I. II	7.82	(5.42–11.4)	< 0.01	2.07	(1.08–3.94)	0.028	2.07	(1.07–3.93)	0.029
Depth of myometrial invasion	≥1/2 vs. < 1/2	3.84	(2.66–5.60)	< 0.01	1.59	(0.99–2.58)	0.054	1.57	(0.97–2.55)	0.061
Lymphovascular invasion	Yes vs. No	3.84	(2.62–5.75)	< 0.01	1.94	(1.16–3.25)	0.01	1.94	(1.17–3.25)	0.01
Cervical invasion	Yes vs. No	2.23	(1.50–3.25)	< 0.01	1.44	(0.92–2.24)	0.10	1.45	(0.92–2.45)	0.10
Ovarian metastasis	Yes vs. No	7.48	(4.68–11.5)	< 0.01	1.21	(0.67–2.13)	0.51	1.21	(0.67–2.14)	0.50
Lymph node metastasis	Yes vs. No	5.64	(3.68–8.58)	< 0.01	1.26	(0.70–2.31)	0.44	1.25	(0.69–2.30)	0.45
	Yes vs. Unevaluated	1.94	(1.20–3.14)	< 0.01	1.27	(0.71–2.31)	0.41	1.27	(0.70–2.31)	0.41
	Unevaluated vs. No	2.91	(1.84–4.51)	< 0.01	0.96	(0.57–1.68)	0.96	0.98	(0.57–1.67)	0.96
Distant metastasis	Yes vs. No	12.5	(8.14–18.9)	< 0.01	5.89	(3.11–11.1)	< 0.01	5.87	(3.10–11.1)	< 0.01
Peritoneal cytology	Positive vs. Negative	4.42	(3.04–6.38)	< 0.01	1.59	(0.98–2.58)	0.058	1.59	(0.97–2.57)	0.06
Adjuvant therapy	Yes vs. No	2.55	(1.71–3.89)	< 0.01	0.45	(0.26–0.78)	< 0.01	0.45	(0.26–0.78)	< 0.01
Mesothelin expression	Yes vs. No	2.74	(1.91–3.94)	< 0.01	2.14	(1.45–3.16)	< 0.01			
CA125 expression	Yes vs. No	1.01	(0.68–1.56)	0.92						
Co-expression of mesothelin and CA125	Yes vs. No	2.86	(2.00–4.12)	< 0.01				2.19	(1.49–3.23)	< 0.01

*CI* confidence interval, *HR* hazard ratio, *CA125* cancer antigen 125, *other carcinomas* carcinomas including serous carcinoma, clear cell carcinoma, carcinosarcoma, and mixed carcinoma, *FIGO* International Federation of Obstetrics and Gynecology

**Table 5:** Univariate analyses and Multivariate analyses for overall survival in patients with endometrial carcinoma about clinicopathological features and mesothelin, CA125 expressions

Variables		Univariate analysis			Multivariate analysis for mesothelin expression			Multivariate analysis for co-expression of mesothelin and CA125		
		HR	(95% CI)	*p*-value	HR	(95% CI)	*p*-value	HR	(95% CI)	*p*-value
Age	≥ 60 vs. < 60	2.01	(1.23–3.35)	< 0.01	1.56	(0.91–2.70)	0.10	1.55	(0.91–2.70)	0.10
Histological type	Grade 2 endometrioid carcinoma vs. Grade 1 endometrioid carcinoma	2.92	(1.36–6.39)	< 0.01	1.46	(0.63–3.42)	0.36	1.47	(0.63–3.45)	0.36
	Grade 3 endometrioid carcinoma vs. Grade 1 endometrioid carcinoma	5.30	(2.46–11.6)	< 0.01	3.68	(1.58–8.78)	< 0.01	3.70	(1.58–8.82)	< 0.01
	Other carcinomas vs. Grade 1 endometrioid carcinoma	8.22	(4.22–17.0)	< 0.01	2.97	(1.34–6.87)	< 0.01	2.99	(1.35–6.91)	< 0.01
FIGO stage	III.IV vs. I.II	7.05	(4.35–11.6)	< 0.01	1.62	(0.64–3.98)	0.29	1.61	(0.64–3.95)	0.30
Depth of myometrial invasion	≥1/2 vs. < 1/2	3.59	(2.22–5.92)	< 0.01	1.47	(0.78–2.80)	0.23	1.45	(0.77–2.78)	0.24
Lymphovascular invasion	Yes vs. no	3.96	(2.37–6.87)	< 0.01	2.47	(1.25–4.90)	< 0.01	2.46	(1.25–4.89)	< 0.01
Cervical invasion	Yes vs. no	2.11	(1.25–3.46)	< 0.01	1.34	(0.73–2.40)	0.33	1.34	(0.73–2.41)	0.33
Ovarian metastasis	Yes vs. no	10.3	(5.95–17.3)	< 0.01	1.75	(0.86–3.57)	0.12	1.75	(0.86–3.57)	0.12
Lymph node metastasis	Yes vs. no	4.50	(2.47–8.04)	< 0.01	1.15	(0.52–2.62)	0.72	1.15	(0.51–2.61)	0.72
	Yes vs. unevaluated	1.09	(0.58–2.01)	0.77	0.92	(0.43–1.97)	0.84	0.92	(0.43–1.97)	0.84
	Unevaluated vs. no	4.11	(2.34–7.15)	< 0.01	1.24	(0.62–2.44)	0.52	1.24	(0.62–2.43)	0.53
Distant metastasis	Yes vs. no	13.8	(8.14–22.8)	< 0.01	5.47	(2.45–12.4)	< 0.01	5.45	(2.44–12.4)	< 0.01
Peritoneal cytology	Positive vs. negative	5.18	(3.20–8.36)	< 0.01	1.93	(1.01–3.66)	0.046	1.92	(1.00–3.65)	0.047
Adjuvant therapy	Yes vs. no	2.00	(1.20–3.43)	< 0.01	0.26	(0.12–0.54)	< 0.01	0.26	(0.12–0.54)	< 0.01
Mesothelin expression	Yes vs. no	3.14	(1.95–5.13)	< 0.01	2.18	(1.31–3.71)	< 0.01			
CA125 expression	Yes vs. no	1.39	(0.79–2.60)	0.25						
Co-expression of mesothelin and CA125	Yes vs. no	3.26	(2.03–5.33)	< 0.01				2.22	(1.33–3.77)	< 0.01

*CI* confidence interval, *HR* hazard ratio, *CA125* cancer antigen 125, *other carcinomas* carcinomas including serous carcinoma, clear cell carcinoma, carcinosarcoma, and mixed carcinoma, *FIGO* International Federation of Obstetrics and Gynecology

Second, we examined the relationship between CA125 expression and the clinical features. No significant associations were noted between CA125 expression and the clinicopathological factors (Table 3). In addition, PFS and OS were not different between the two groups according to CA125 expression (Fig. 2c: PFS, *p* = 0.97; Fig. 2d: OS, *p* = 0.64).

Thirdly, Analysis to compare patients without both mesothelin and CA 125 expression with other patients was performed. There were no statistical significances of several clinicopathological features, PFS (*p* = 0.83), and OS (*p* = 0.76) between two groups.

Finally, the relationship between mesothelin and CA125 co-expression and clinicopathological factors was examined. Patients in the group with positive co-expression had myometrial invasion of more than 50% (*p* = 0.016) and positive lymphovascular invasion (*p* = 0.02) compared with those with negative co-expression (Table 3). Patients with positive co-expression had worse PFS (Fig. 2d: PFS, *p* < 0.01) and OS (Fig. 2e: OS, *p* < 0.01) than those with negative co-expression. Multivariate analyses for PFS and OS demonstrated that positive co-expression was a worse prognostic factor for PFS (Table 4: HR = 2.19, 95% CI = 1.49–3.23; *p* < 0.01) for OS (Table 5: HR = 2.22, 95% CI = 1.33–3.77, *p* < 0.01).

## Discussion

In our study, mesothelin expression and co-expression of mesothelin and CA125 were more often observed in patients with other histological types – including serous carcinoma, clear cell carcinoma, and carcinosarcoma –mixed carcinoma, and associated deeper myometrial invasion and lymphovascular invasion. Furthermore, multivariate analysis demonstrated that mesothelin expression and co-expression of mesothelin and CA125 were independent prognostic factors.

Mesothelin expression and co-expression of mesothelin and CA125 were associated with tumor aggressiveness in several types of carcinomas [7–15]. In endometrial carcinoma, serous carcinoma, clear cell carcinoma, carcinosarcoma, and mixed carcinoma are more aggressive histological subtypes than grade 1 and 2 endometrioid carcinoma [22–25]. In addition, the depth of myometrial invasion and lymphovascular invasion were the factors associated with tumor aggressiveness and worse prognosis [26–28]. The relationships between mesothelin expression and co-expression and clinicopathological features in our study were related to these facts. Evaluating for mesothelin expression and co-expression of mesothelin and CA125 might be useful for the detection of aggressive subtypes of endometrial carcinomas.

Our previous reports demonstrated co-expression was associated with tumor malignancy such as lymphovascular invasion and lymph node metastasis in serous carcinoma [20]. In our current study, co-expression was associated with tumor aggressiveness. Mesothelin and co-expression might be the common important factors to enhance malignant potential regardless of histological subtypes.

Mesothelin independently activate epithelial-to-mesenchymal transition and tumor progression in some malignant tumors [29]. Overexpression of mesothelin alone could constitutively activate the nuclear factor-kappa B, mitogen-activated protein kinase, and phosphoinositide 3-kinase intracellular pathways, promoting cell proliferation and resistance to apoptosis [30]. Besides, mesothelin-accelerated tumor progression is accelerated by CA125 [6, 11]. In our study, almost all patients with positive mesothelin were positive for CA125 expression, which indicated that mesothelin might play an important role in the development of ECs. Also, the association that almost all patients with positive mesothelin were positive for CA125 expression was observed in pancreatic carcinoma [10]. There was the possibility that only CA125 positive cancer cell could stabilize mesothelin expression and CA125-mesothelin complex. However, because it was just a hypothesis, we considered further study to examine the fact.

Only mesothelin expression was associated with worse prognosis in several carcinomas [7–9, 12–15]. Moreover, co-expression was correlated with worse prognosis in pancreatic, ovarian, and endometrial serous carcinomas [10, 11, 20]. On the other hand, mesothelin expression was better prognosis factor in gastric carcinoma, ovarian carcinoma, and mesothelioma [31–33]. Clinical significances of mesothelin might be different among several carcinomas. In our study, only mesothelin expression and co-expression of mesothelin and CA125 were worse prognostic factors in several histological subtypes of endometrial carcinoma. Therefore, mesothelin may be a potential candidate for a new target therapy in endometrial carcinomas.

Mesothelin have been studied as a biomarker for targeted therapy including antibody-based drugs, cancer vaccine, and chimeric antigen receptor T cell therapies in several types of solid tumors [34, 35]. In phase I study for mesothelin targeted therapies, 2.4 mg/kg of mesothelin-targeting antibody-based drug administered three times a week showed safety and partial responses in 3 of 10 (30%) patients with platinum-resistant ovarian cancer [36]. Furthermore, the in vivo proliferation of human uterine -cancer cells with mesothelin expression was inhibited by treatment with a mesothelin-targeting antibody-based drug [37]. In our study, mesothelin were used to determine patients with worse prognosis. Therefore, using mesothelin as a biomarker by immunohistochemical analysis, mesothelin-targeted therapies might be helpful in the treatment of EC.

This study has some limitations. It was performed in a single institution and was retrospective in nature. Our study could not include all patients during observational periods. Among a total of 675 patients during observational periods, 130 patients without our surgical specimen and 60 patients without clinical information were excluded. Finally, 485 patients were included in our study. Our study included selection bias to some extent but minimized it because the reason of exclusion was not our intention. Also, there were several confounding factors associated with PFS and OS. However, in our study, multivariate analysis was performed. In results, mesothelin and co-expression was identified as the independent prognostic factors. Therefore, we considered our study could minimized the effect of confounding factors. However, the strength point of our study included relatively large samples. Our study showed that mesothelin and co-expression of mesothelin and CA125 might be important factors in EC.

## Conclusions

Co-expression mesothelin and CA125 might be a better biomarker for predicting the prognosis of EC. Only mesothelin expression might be the better biomarker to predict the prognosis. Further large-scale studies are needed to confirm these findings.

## Data Availability

All data analyzed in this study are available from the corresponding author upon reasonable request.

## Abbreviations

CA: Cancer antigen
EC: Endometrial carcinoma
OS: Overall survival
PFS: Progression free survival
TMA: Tissue microarray
HR: Hazard ratio
